# SOX9 promotes the invasion and migration of lung adenocarcinoma cells by activating the RAP1 signaling pathway

**DOI:** 10.1186/s12890-023-02740-w

**Published:** 2023-11-02

**Authors:** Jun-fa Yang, Qing Liao, Chen-lin Lu

**Affiliations:** Department of Respiratory and Critical Care Medicine, Taizhou School of Clinical Medicine, The Affiliated Taizhou People’s Hospital of Nanjing Medical University, Nanjing Medical University, Taizhou, 225300 Jiangsu China

**Keywords:** Lung adenocarcinoma (LUAD) cell, SOX9, RAP1 signaling pathway, Invasion, Migration

## Abstract

**Objective:**

SOX9 has been shown to be related to the metastasis of various cancers. Recently, it has been reported that SOX9 plays a regulatory role in lung adenocarcinoma (LUAD) cell metastasis, but the specific mechanism remains to be explored. Therefore, the objective of this study was to observe the effect and mechanism of SOX9 on the invasion and migration of LUAD cells.

**Methods:**

RT-qPCR was applied to observe the expression of SOX9 and RAP1 in tumor tissues and corresponding normal lung tissues collected from LUAD patients. Co-immunoprecipitation and Pearson correlation to analyze the expression correlation of SOX9 with RAP1. To observe the role of SOX9, the invasion and migration levels of LUAD A549 cells in each group were observed by Transwell invasion assay and Scratch migration assay after knocking down or overexpressing SOX9. Besides, the expression levels of RAP1 pathway-related proteins (RAP1, RAP1GAP and RasGRP33) were observed by RT-qCPR or western blot. Subsequently, RAP1 was overexpressed and SOX9 was knocked down in A549 cells, and then the cell invasion/migration level and RAP1 pathway activity were assessed.

**Results:**

The expression levels of SOX9 and RAP1 in tumor tissues and A549 cells of LUAD patients were significantly increased and positively correlated. Overexpression of SOX9 or RAP1 alone in A549 cells enhanced the invasion and migration ability of cells, as well as up-regulated the expression levels of RAP1, RAP1GAP and RasGRP33. However, knocking down SOX9 decreased cell invasion and migration levels and weakened the activity of RAP1 pathway. Notably, overexpressing RAP1 while knocking down SOX9 significantly activated RAP1 pathway and promoted cell invasion and migration.

**Conclusion:**

Overexpression of SOX9 in LUAD can significantly activate the RAP1 signaling pathway and promote cell invasion and migration.

**Supplementary Information:**

The online version contains supplementary material available at 10.1186/s12890-023-02740-w.

## Introduction

Lung cancer is a well-known fatal malignancy and the main cause of cancer-related death [1]. Currently, lung cancer has become the second most prevalent cancer in both men and women, accounting for 25% of all cancer-related deaths [2]. More terribly, the incidence of lung cancer still remains high. In 2020, 220,600 new cases of lung cancer and 179,600 deaths were reported worldwide [3]. Moreover, the survival rate of lung cancer patients is not high because they are diagnosed at an advanced stage. During 2007–2013, the 5-year survival rate of lung cancer patients was only 18% [4]. There are various types of lung cancer, such as non-small cell lung cancer (NSCLC) that accounts for about 85% of all lung cancers. Lung adenocarcinoma (LUAD) is the most common histological subtype of NSCLC [5]. At present, LUAD treatments mainly include surgical resection, chemotherapy, radiationtherapy and immunotherapy. Despite advances in these treatments, the overall survival rate (OS) nor the mortality rate of LUAD has been improved, mainly owing to the lack of effective therapeutic targets [6]. Furthermore, it cannot be ignored that surgical resection is the only cure for LUAD, and that LUAD cannot be treated if distant metastasis has taken place [7]. Therefore, it is urgent to reveal the molecular mechanism of LUAD metastasis and identify effective biomarkers and therapeutic targets.

SOX9, belonging to the SOX group, has three domains [8], including a DNA binding domain with a high mobility group box [9] and a transactivation domain that plays a crucial role in embryonic development [10]. Studies have shown that SOX9 contributes significantly to cell lineage limitation and terminal differentiation through specific time and space expression patterns [8]. According to a recent study, the imbalance of SOX9 expression is a driving factor for the occurrence and development of cancer and a boosting factor for the malignant growth of cells under different conditions [11]. But whether SOX9 functions specifically as an oncogene [12] or a tumor suppressor [13] is a matter of some debate. Some studies have pointed out that the expression level of SOX9 is correlated with tumor invasion, metastasis and patient survival [14, 15]. Interestingly, Huang et al. discovered that SOX9 overexpression was not only significantly associated with tumor (T, P = 0.03), node (N, P = 0.000), and metastasis (M, P = 0.032) categories in clinical samples of NSCLC, but also significantly promoted the migration, invasion and epithelial-mesenchymal transition (EMT) of NSCLC cells [16]. Besides, Pei et al. discovered that the long non-coding RNA rhabdomyosarcoma 2-associated transcript (lncRNA-RMST) could promote the ubiquitination of SOX9 protein, thereby inhibiting the growth and metastasis of LUAD [17]. Current investigations have revealed the importance of SOX9 in the development of LUAD. For example, Capaccione et al. proposed that SOX9 may be used as a hub to mediate crosstalk across key carcinogenic pathways in LUAD [18]. However, the mechanism by which SOX9 regulates the progress of LUAD has not been fully uncovered. In addition, RAP1 pathway is considered as a crucial signaling pathway for regulating tumor metastasis [19]. The relevance of this pathway in LUAD has, however, received relatively little research, and only some studies have mentioned that the target gene of exosomes in pleural effusion of patients with LUAD may be related to RAP1 [20]. The relationship between SOX9 and RAP1 is not clear, either. Therefore, this study made an attempt to explore the regulatory correlation between SOX9 and the RAP1 signaling pathway as well as their effects on LUAD metastasis, thereby providing new insights for revealing the mechanism of LUAD metastasis.

## Materials and methods

### Clinical sample collection

A total of 40 samples including tumor tissue samples (Tumor group 20) and matched normal lung tissue (Normal group 20) samples were collected from LUAD patients who received histopathology diagnosis in The Affiliated Taizhou People’s Hospital of Nanjing Medical University, Taizhou School of Clinical Medicine, Nanjing Medical University from December 2021 to January 2023 and then stored at -80℃. The inclusion criteria were as follows: patients (1) diagnosed as LUAD pathologically; (2) aged from 18 to 75 years old; (3) who never received any treatment before participating in this study; (4) who volunteered to participate in this study; (5) informed of the contents of this study and providing written consent. The exclusion criteria: patients (1) participating in other clinical studies at the same time; (2) with any other cancers in the past 5 years or diagnosed during screening; (3) with active brain metastases [21]. This study was conducted according to the Declaration of Helsinki and clinical practice guidelines, and the agreement was given by the local ethics committees of the participating centers.

### Cell culture and treatment

Both human LUADA549 cells and human normal lung epithelium BEAS-2B cells were purchased from National Collection of Authenticated Cell Cultures. BEAS-2B and A549 cells were separately cultured in a Dulbecco’s Modified Eagle Medium (DMEM) and a Roswell Park Memorial Institute (RPMI) 1640 Medium at 5%CO_2_ under 37 °C, both of which contained 10% fetal bovine serum (FBS) and 1% penicillin/streptomycin. Subsequently, pCMVp-NEO-BAN vector with SOX9 and RAP1 overexpression and shRNA-SOX9 expression was transfected into A549 cells (SOX9, RAP1 and sh-SOX9) by FuGENE® 6 transfection reagent (E2691, promega, USA). Besides, A549 cells were transfected with shRNA-SOX9 and RAP1 overexpression vectors. After 72 h of cell culture under conditions of 37 °C and 5% CO_2_, the next experiment was performed. In this study, shRNA-SOX9 vector and its negative control vector (sh-NC), and SOX9 overexpression vector and its negative control vector were all synthesized by Guangzhou Tsingke Biotech Co., Ltd.

### RT-qPCR

The separation of total RNA from lung tissue samples was carried out under the instructions of the RNAprep Pure Tissue Kit (DP431, TIANGEN). As for the RNA extraction from A549 cells, the transfected cells were placed on ice at first, then the cell culture solution was removed, the cells were washed three times using pre-cooled PBS, and finally the total RNA of cells was isolated and purified by RNA Easy Fast Kit (DP451, TIANGEN). The concentration of RNA was determined by NanoDrop spectrophotometers (840-317400, ThermoFisher, USA). Upon diluting the RNA concentration to 500 ng/µL, 2 µg of RNA was reversely transcribed into complementary deoxyribonucleic acid (cDNA) according to the instructions of FastKing cDNA First Chain Synthesis Kit (KR116, TIANGEN). Finally, the expression levels of SOX9, RAP1 and GAPDH (an internal control) in tissue or cell samples were detected by RealUniversal Color PreMix (FP201, TIANGEN). Based on the acquired data, the relative expression of the gene was calculated using 2 ^−ΔΔCt^ method. The primer sequences shown in Table 1 were synthetized by Sangon Biotech (Shanghai) Co., Ltd.

**Table 1:** Primer sequences

Gene name	Sequences (5’ to 3’)
SOX9	F: AGGAAGCTCGCGGACCAGTAC
	R: GGTGGTCCTTCTTGTGCTGCAC
RAP1	F: ACTTACAGGACCTGAGGGAACAG
	R: CCTGCTCTTTGCCAACTACTCG
GAPDH	F: GTCTCCTCTGACTTCAACAGCG
	R: ACCACCCTGTTGCTGTAGCCAA

### Transwell invasion assay

Transwell invasion assay was adopted to observe the invasion of cells in each group. Firstly, the matrix gel and the culture medium were fully mixed and coated in the upper chamber. Then, A549 cells were seeded in a 6-well plate at a density of 5 × 10^6^ cells/well. After complete adherence to the wall, the cells were subjected to a 72-h transfection. Next, the cells were resuspended with trypsin, and then introduced in the upper chamber at a density of 5 × 10^5^ cells/well. And the culture medium containing 10% FBS was added into the lower chamber. Following 24 h of cell invasion, the lower chamber was fixed with 4% paraformaldehyde and then stained with 0.05% crystal violet. Images of cell invasion were captured by a microscope, a representative area was selected for photographing, and the IMAGE-J software was employed for quantification. Finally, cell invasion in each group was observed.

### Scratch migration assay

The migration of A549 cells in different groups was measured by scratch migration assay in vitro. After transfection for 72 h, A549 cells were collected and seeded into 6-well plates at a density of 5 × 10^6^ cells/well. An overnight adherence was allowed for the cell confluence of 80%. Monolayer cells were scratched vertically with a 10µL pipette tip, and the dropped cells were gently washed with PBS. Next, the wound healing image was captured by a Nikon inverted microscope at the specified time point. The gap width was calculated using GraphPad Prism software, the healing area was measured, and the cell movement was calculated according to the healing percentage.

### Western blot

After transfection, A549 cells were collected and the culture medium was removed. Next, the cells were washed with precooled PBS for three times. Following that, the cells were supplemented with 1 ml of RIPA buffer (Beyotime) containing protease inhibitors, and then transferred into a centrifuge tube. Subsequently, the cells were ultrasonically broken for 3 min under the conditions of a 3-s stop for every 2-s work. Subsequent to a 10-min cell centrifugation at 4 °C and 12,000 g, the concentration of total protein in cells of each group was determined by a BCA protein assay kit (Beyotime). Next, 20 µg of total protein was mixed with 5× SDS loading buffer and then boiled for 10 min. Later, the protein was separated by 10% SDS-PAGE, and the target protein was transferred to polyvinylidene fluoride (PVDF) membranes by electrotransfer. Following a 2-h incubation with 5% skim milk, the target protein was incubated with primary antibodies at 4 °C overnight. The used primary antibodies included RAP1 (1:1000, ab272863, Abcam), RAP1GAP (1:1000, ab32373, Abcam), RasGRP3 (1:1000, ab124823, Abcam). Then, the membranes were incubated with goat anti-rabbit or mouse secondary antibody for 1 h. The enhanced chemiluminescence (SuperSignal ECL, ThermoFisher) was applied to develop proteins in the membranes. The expression level of each protein was observed through Gel Doc XR + Gel Documentation System (Bio-red, USA).

### Co-immunoprecipitation (CO-IP)

RIPA buffer (Beyotime Biotechnology) containing a protease inhibitor (Roche Applied Science, USA) was added to the transfected A549 cells, which were later placed on a low-speed rotating shaker at 4 °C for a 30-min lysis. The lysate was then centrifuged at 12,000 rpm at 4 °C for 10 min. After the centrifugation, the supernatant was collected and part of the lysate was removed as an input sample. The remaining supernatant were shaken with specific primary antibody overnight on a rotating shaker at 4 °C. Protein A/G plus-agarose (sc-2003, SantaCruz Biotechnology, USA) was then added to the mixture and incubated in a rotating shaker at 4 °C for 6 h. Finally, immunoprecipitates and whole cell lysates were collected for western blot analysis.

### Statistics and analysis

SPSS16.0 software was employed for data analysis, and GraphPad Prism 9.2.0 software for drawing. Variance analysis was adopted for comparisons between groups, t test for two independent samples, and one-way ANOVA for more than two independent samples. Additionally, Pearson analysis was performed to clarify the correlation between SOX9 and RAP1 expression in clinical samples. P < 0.05 indicated a significant difference.

## Results

### Up-regulation of SOX9 expression in lung adenocarcinoma

**Fig. 1:**
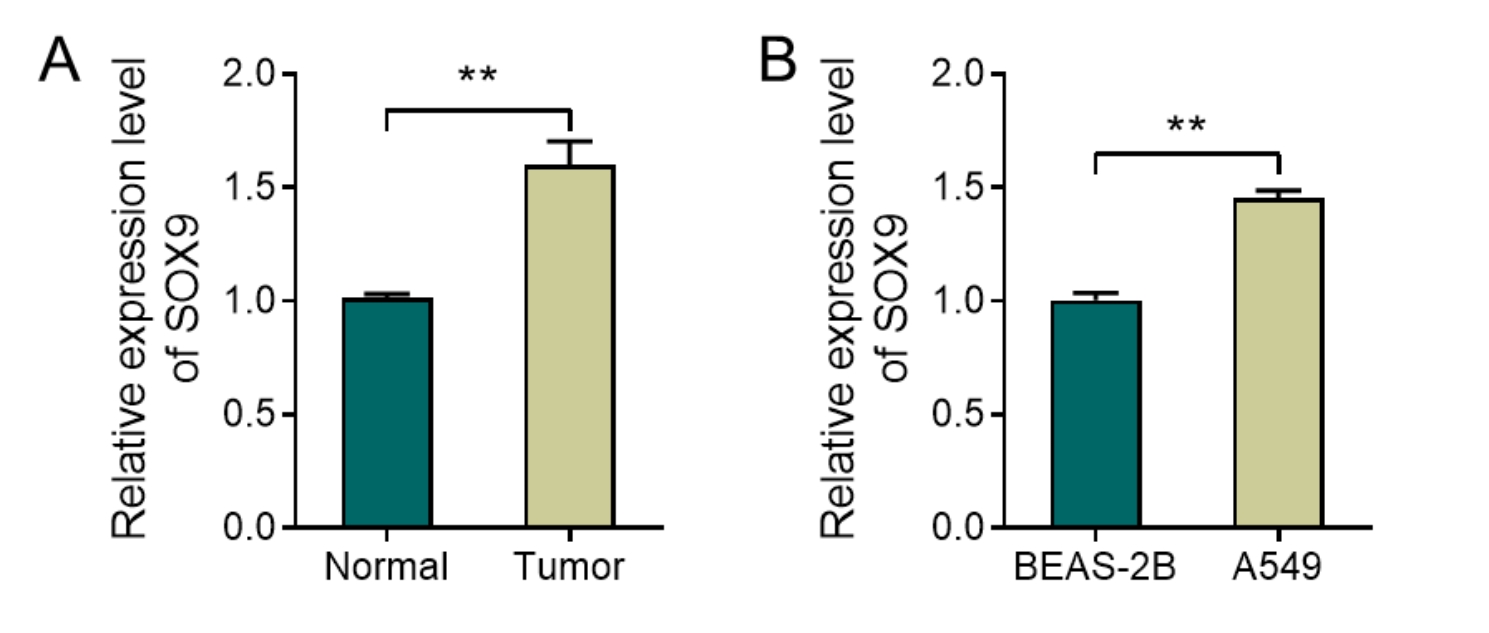
Up-regulation of SOX9 expression in lung adenocarcinoma. A-B, RT-qPCR to detect the expression level of SOX9 in clinical samples (**A**) and cells (**B**). **P < 0.01

The change of gene expression is one of the factors that affect the development of diseases. Therefore, the expression changes of SOX9 in LUAD were first observed in this study. Based on the RT-qPCR results, it was evident that the SOX9 expression level was significantly increased in the Tumor group compared with that in the Normal group in the collected clinical samples (Fig. 1A, P < 0.05). Notably, the SOX9 expression level was also up-regulated in human LUAD A549 cells relative to that in human normal lung epithelium BEAS-2B cells (Fig. 1B, P < 0.05). Therefore, changes in the SOX9 expression level may play a key role in the development of LUAD.

### Knockdown of SOX9 inhibits A549 cell invasion and migration

**Fig. 2:**
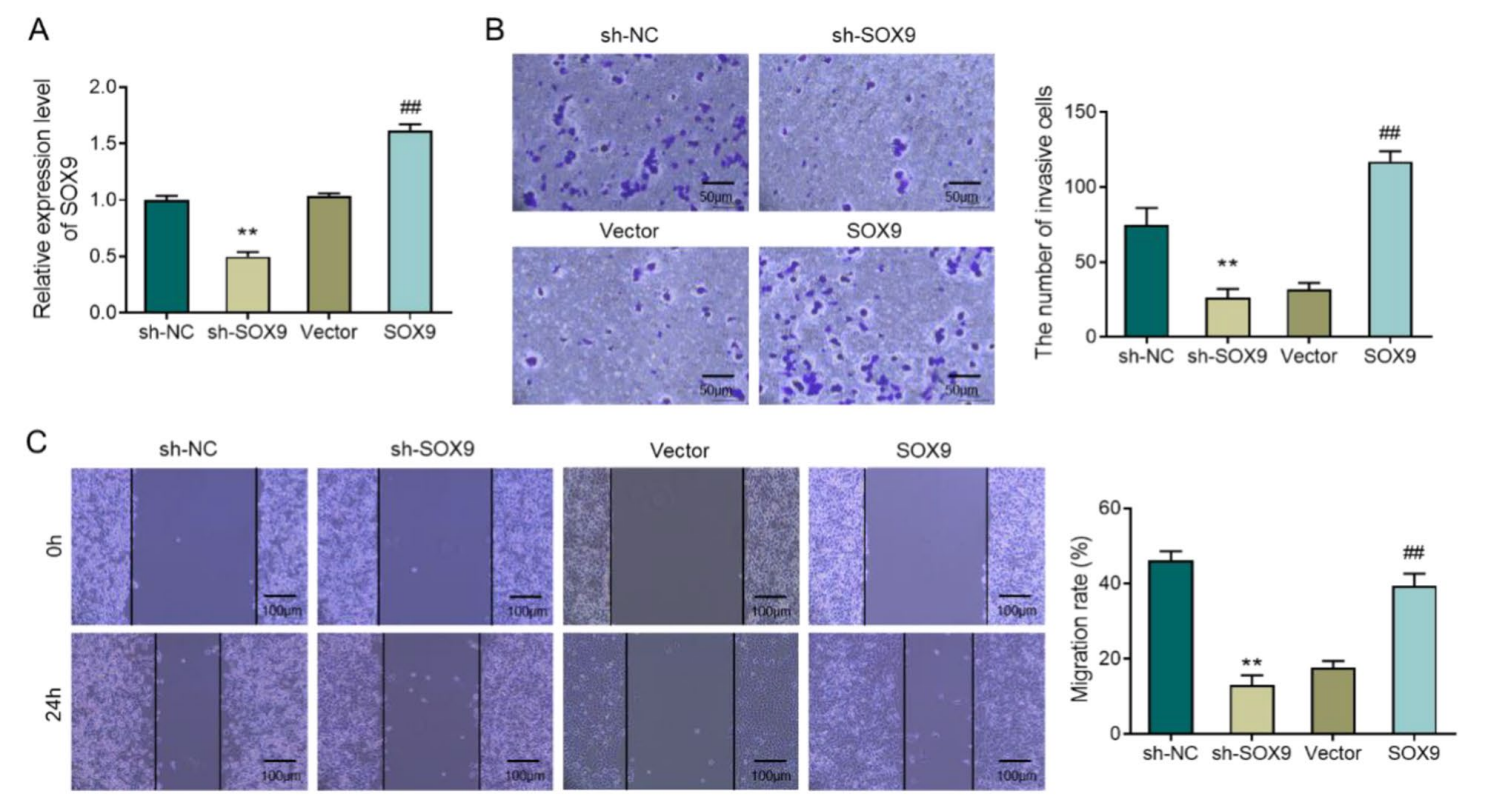
Knocking down SOX9 inhibits invasion and migration of A549 cells. **A**, RT-qPCR to detect the expression level of SOX9 in cells of each group; **B**, Transwell to observe the invasion level of cells in each group; **C**, Scratch migration assay to check the migration ability of cells in each group. **P < 0.01, vs. sh-NC; ##P < 0.01, vs. Vector

To explore the role of SOX9 in LUAD, changes in cell invasion and migration were observed after knocking down or overexpressing SOX9 in A549 cells. The results of RT-qPCR showed that knocking down SOX9 could significantly reduce the expression level of SOX9 in A549 cells in contrast to the sh-NC group. Moreover, compared with the Vector group, the overexpression vector of SOX9 could significantly raise the expression level of SOX9 in cells (Fig. 2A, P < 0.05). Subsequently, the invasion and migration ability of cells were observed by Transwell and Scratch assays. The assay results showed that in comparison with the sh-NC group, the invasion and migration ability of A549 cells in the sh-SOX9 group were weakened (Fig. 2B-C, P < 0.05). However, compared with the Vector group, the invasion and migration ability of A549 cells in the SOX9 group were enhanced (Fig. 2B-C, P < 0.05). In short, suppression of SOX9 may be a potential strategy to suppress LUAD metastasis.

### Up-regulation of SOX9 in lung adenocarcinoma activates the RAP1 signaling pathway

**Fig. 3:**
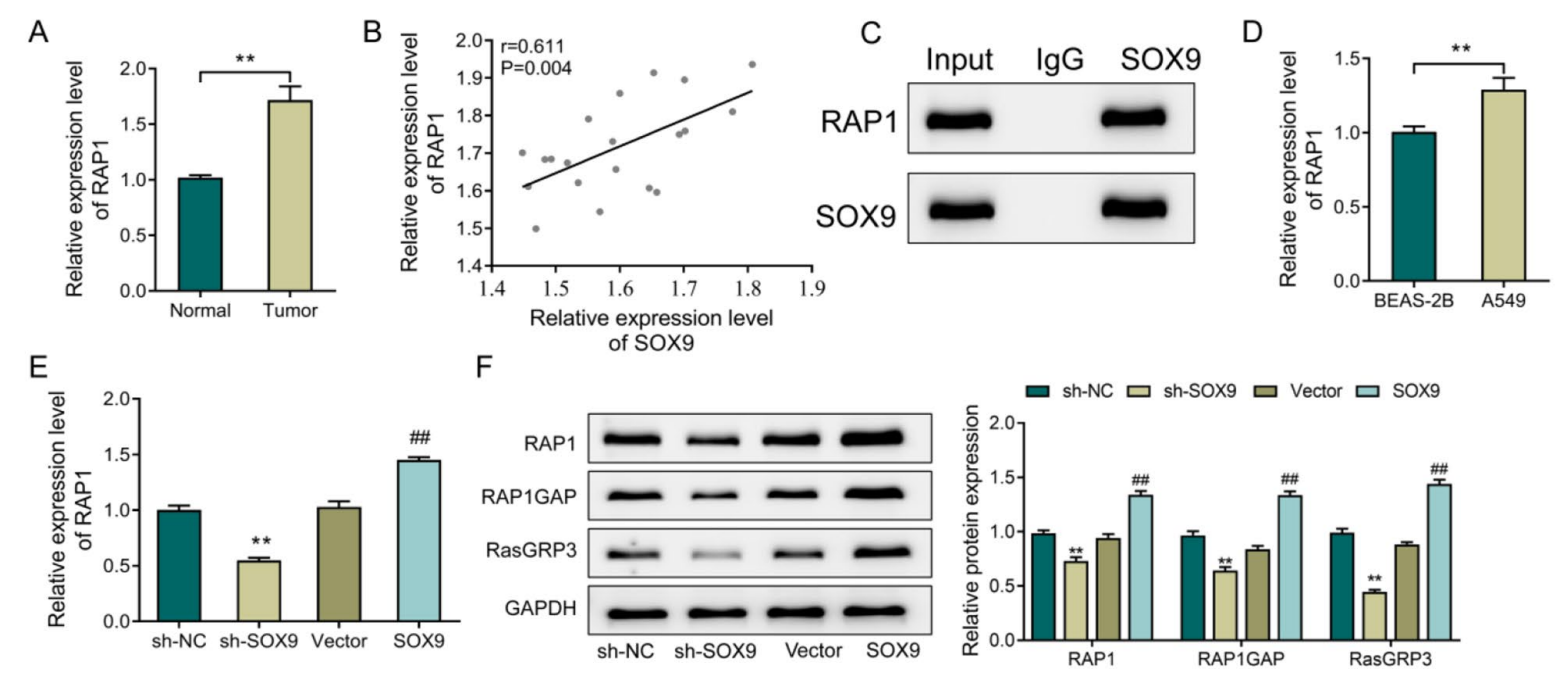
Up-regulating SOX9 in lung adenocarcinoma activates the RAP1 signaling pathway. **A**, RT-qPCR to check the expression of RAP1 in clinical samples; **P < 0.01; **B**, Pearson analysis for the correlation between SOX9 and RAP1 expression in clinical samples; **C**, CO-IP is used to analyze the interaction between RAP1 and SOX9. D-E, RT-qPCR to measure the expression of RAP1 in BEAS-2B cells, A549 cells (**D**) **P < 0.01;, and different expression levels of SOX9 cells (**E**);**P < 0.01, vs. sh-NC; ##P < 0.01, vs. Vector; **F**, Western blot to detect the expression levels of RAP1, RAP1GAP and RasGRP3 in each group of cells. **P < 0.01, vs. sh-NC; ##P < 0.01, vs. sh-SOX9.

To reveal the mechanism by which SOX9 controls LUAD metastasis, the expression level of RAP1 was observed in clinical samples. The observation results demonstrated a much higher expression level of RAP1 in the Tumor group compared with the Normal group (Fig. 3A, P < 0.05). Then, according to Pearson correlation analysis, the expression of RAP1 was positively correlated that of SOX9 in LUAD tissues (Fig. 3B, P = 0.004). The CO-IP results further indicated the interaction between RAP1 and SOX9 (Fig. 3C). Relative to BEAS-2B cells, the expression level of RAP1 was up-regulated in A549 cells (Fig. 3D-E, P < 0.05). Moreover, in comparison with the NC group, sh-SOX9 could inhibit the mRNA and protein levels of RAP1 while decreasing the protein levels of RAP1GAP and RasGRP3. As opposed to the Vector group, overexpression of SOX9 could up-regulate the mRNA and protein levels of RAP1 as well as the protein levels of RAP1GAP and RasGRP3 (Fig. 3F, P < 0.05). All in all, up-regulating SOX9 in LUAD could activate the RAP1 signaling pathway.

### SOX9 regulates the invasion and migration of A549 cells by activating the RAP1 signaling pathway

**Fig. 4:**
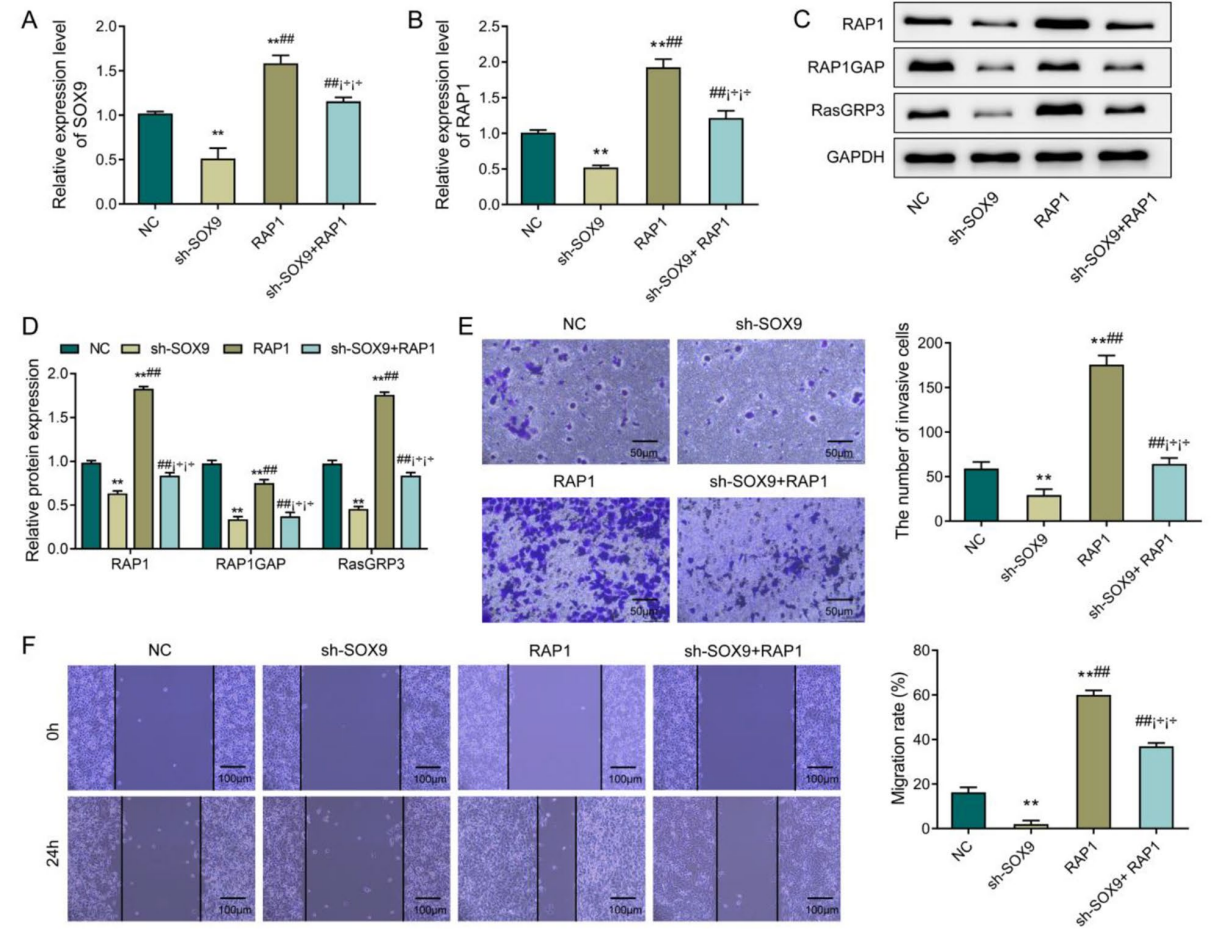
SOX9 regulates the invasion and migration of A549 cells by activating the RAP1 signaling pathway. **A**-**B**, RT-qPCR to test the expression levels of SOX9 and RAP1 in cells of each group; **C**-**D**, Western blot to detect the expression levels of RAP1, RAP1GAP and RasGRP3 in cells; **E**, Transwell to observe cell invasion levels; **F**, Scratch migration assay to observe the migration ability of cells. **P < 0.01, vs. sh-NC; ##P < 0.01, vs. sh-SOX9; ¡÷¡÷P < 0.01, vs. RAP1.

To further verify the role and correlation of SOX9 and RAP1 in LUAD, SOX9 was knocked down and RAP1 was overexpressed in A549 cells. The results of RT-qPCR revealed that the mRNA expression levels of SOX9 and RAP1 in the sh-SOX9 + RAP1 group were higher than those in the sh-SOX9 group. Compared with the RAP1 group, the sh-SOX9 + RAP1 group showed a significant drop in levels of SOX9 and RAP1 (Fig. 4A-B, P < 0.05). In terms of the western blot results, a notable rise was observed in the expression levels of RAP1, RAP1GAP and RasGRP3 in the sh-SOX9 + RAP1 group compared with the sh-SOX9 group; the sh-SOX9 + RAP1 group exhibited lower protein expression levels of RAP1, RAP1GAP and RasGRP3 than the RAP1 group (Fig. 4C-D, P < 0.05). Subsequently, the invasion and migration ability of cells were observed by Transwell and Scratch assays. Specifically, compared with the sh-SOX9 group, the sh-SOX9 + RAP1 group displayed increased invasion and migration ability of cells; the invasion and migration ability of A549 cells in the sh-SOX9 + RAP1 group decreased in comparison with the RAP1 group (Fig. 4E-F, P < 0.05). The above findings proved that SOX9 could ehance the invasion and migration ability of A549 cells by activating the RAP1 signaling pathway.

## Discussion

The mining of LUAD biomarkers has been the subject of numerous research reports as of late. For instance, FAM72 has been discovered to serve as a biomarker of poor prognosis in patients with LUAD [7], ERO1L has been reported to be related to the drug resistance of immunotherapy in LUAD patients [22], and lncRNA DGCR5 is considered to be involved in the progress of LUAD [23]. These biomarkers, which are constantly being discovered, provide important clues for the formulation of therapeutic regimen for LUAD. However, the known biomarkers are not enough to improve the poor survival of LUAD patients, hence additional research into biomarkers associated with LUAD development and metastasis is required. In this study, the expression level of SOX9 was significantly increased in LUAD tissues and cells. Moreover, research by Jana et al. showed considerably up-regulated expression level of SOX9 in breast cancer [24]. Carrasco-Garcia et al. discovered that metastatic colorectal cancer cells had higher expression of SOX9 than static colorectal cancer cells [25]. Therefore, we speculated that the up-regulation of SOX9 expression may function as a protooncogene in LUAD. In this paper, after altering the expression level of SOX9 in A549 cells, we observed that high expression of SOX9 significantly increased the migration and invasion levels of cells. Furthermore, Wang et al. disclosed that SOX9 acted as the transcription factor of FOXA1 to significantly promote the tumorigenicity of lung cancer cells [26]. Overexpression of SOX9 can significantly raise the migration and invasion level of H1299 cells, as revealed by previous studies [18, 27, 28]. The expression level of SOX9 was proved again to be closely related to the migration and invasion level of lung cancer cells (A549 cells) in this article.

RAP1, a member of the Ras-like small GTP binding protein family, can bind to guanosine triphosphate (GTP) or guanosine diphosphate (GDP) [29]. Studies have demonstrated that RAP1 activity is positively regulated by guanine nucleotide exchange factors (GEFs) and negatively regulated by GTPase-activating proteins (GAPs) [30]. Compared with non-cancer cells, the activated RAP1 is highly expressed in cancer cells. For example, in breast epithelial cells, increased sugar uptake linduces the malignant phenotype of non-cancerous breast cells via activating integrins through RAP1 up-regulation [31]. Studies have also revealed that the expression of RAP1 in lung cancer is significantly up-regulated, while down-regulation of RAP1 expression can reduce the migration and invasion of NSCLC cell lines [32]. Notably, various drugs or Chinese herbal medicine preparations have been proved to be able to inhibit cancer via directly or indirectly mediating the RAP1 signaling pathway. For instance, Han et al. claimed that He-Chan Pian inhibited the metastasis of NSCLC through the regulation of GREM1/RAP1 signaling pathway mediated by miR-205-5p [33]. Additionally, it’s reported that propofol can regulate the activity of RAP1 pathway, thereby increasing the chemosensitivity of NSCLC cells [34]. Overall, RAP1 plays an important role in the development of LUAD. In this research, the expression level of RAP1 was markedly increased in LUAD tissues and cell lines and positively correlated with the expression of SOX9. On the other hand, overexpression of RAP1 significantly enhanced the invasion and migration ability of LUAD cells. It is logical to speculate that SOX9 may promote cancer through activating RAP1. Besides, after knocking down or overexpressing SOX9 in cells, the expression levels of RAP1 signaling pathway related proteins and RasGRP3 changed with the change of SOX9 expression. Interestingly, the expression level of RAP1GAP also increased with the increase of RAP1 expression. Previous reports on gastric cancer and colorectal cancer reckoned RAP1GAP as a tumor suppressor gene [35, 36]. However, other investigations have demonstrated that RAP1GAP can promote the invasion and migration of squamous cell carcinoma cells and myeloid leukemia cell lines [37, 38]. Hence, RAP1GAP appears to have more complex roles in addition to acting as a tumor suppressor gene. SOX9 was knocked down and RAP1 was overexpressed in cells to investigate their roles in LUAD in this study. The investigation results indicated that the expression level of RAP1GAP also changed in tandem with the RAP1 expression. Moreover, the effect of knocking down SOX9 on the invasion and migration of LUAD cells was reversed by the overexpression of RAP1. Therefore, SOX9 could affect the invasion and migration of LUAD by activating the RAP1 signaling pathway activity. In hepatocellular carcinoma, CD73 has been reported to activate AKT signaling via a Rap1/P110β cascade. Specifically, CD73 activates SOX9 transcription through c-Myc and prevents SOX9 degradation by inhibiting glycogen synthase kinase 3β, promoting SOX9 expression and enhancing its protein stability. These actions are essential for maintaining the traits of CSCs [39]. Therefore, we speculate that SOX9 may be a transcription factor for proteins associated with the RAP1 signaling pathway, or it may play other roles in this signaling pathway.

However, neither the specific mechanism by which SOX9 activates RAP1 nor the reason why RAP1 and RAP1GAP increased at the same time in LUAD cell lines was clarified in this study. It’ s possible put down to the fact that the expression of RAP1GAP decreases accordingly as RAP1 decreases to prevent the decrease of RAP1 in cells. Moreover, excess RAP1GAP mRNA will be also quickly degraded; otherwise, it may lead to other diseases.

In addition, we only performed in vitro experiments and verified our results in a single cell line; we did not further explore the prognosis and expression of SOX9 and RAP1 at different stages of primary tumors and in non-metastatic and metastatic LUAD samples. Further research is needed to make up for these shortcomings in study design.

## Conclusion

To sum up, the up-regulated expression of SOX9 is closely related to the increase of cell invasion and migration ability in patients with LUAD. Furthermore, SOX9 can play a biological role by activating the RAP1 signaling pathway. Therefore, SOX9 may serve as a new biological target for the treatment of LUAD.

### Electronic supplementary material

Below is the link to the electronic supplementary material.

Supplementary Material 1

## Data Availability

The data used to support the findings of this study are available from the corresponding author upon request.
