# Supplementary figures and images for: SOX9 promotes the invasion and migration of lung adenocarcinoma cells by activating the RAP1 signaling pathway
Source: BMC Pulm Med. 2023 Nov 2;23:421. doi: 10.1186/s12890-023-02740-w (PMC10623714; doi:10.1186/s12890-023-02740-w)

Figure 3C

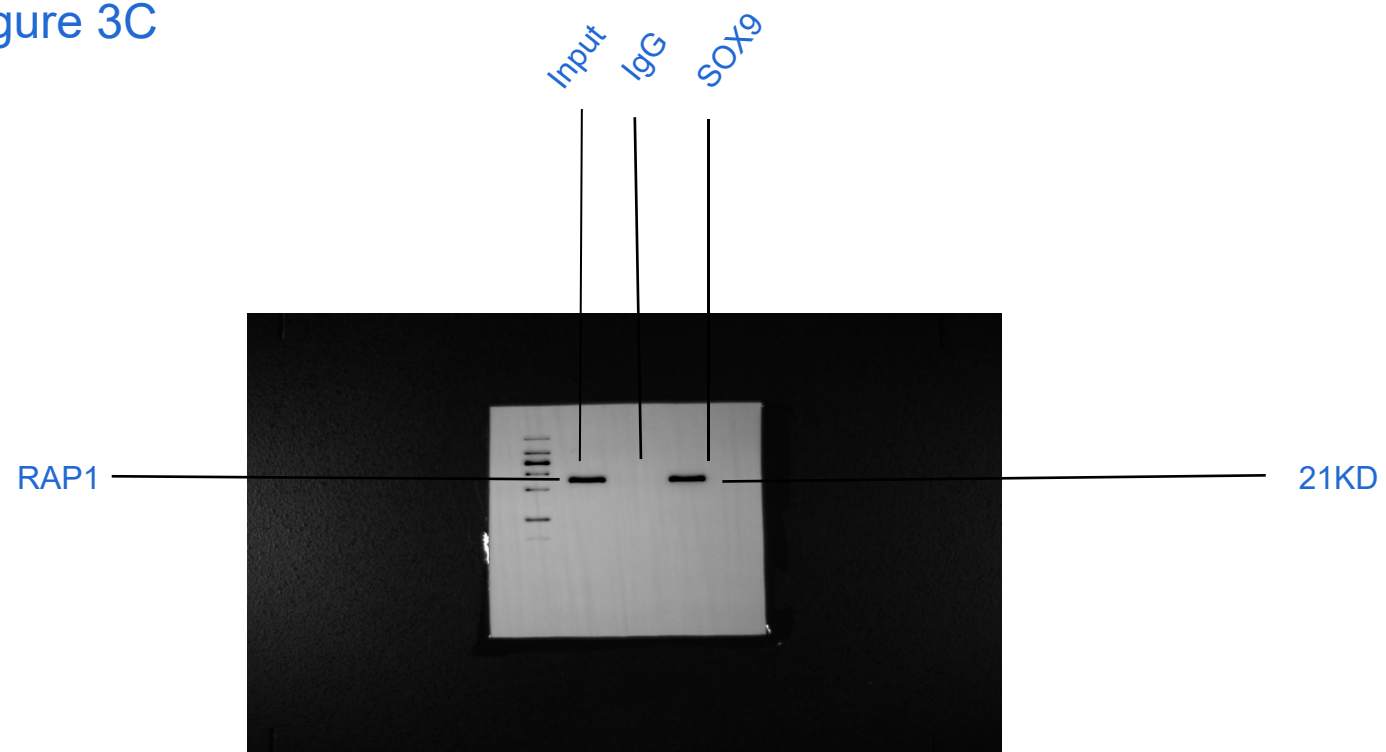

Figure 3C

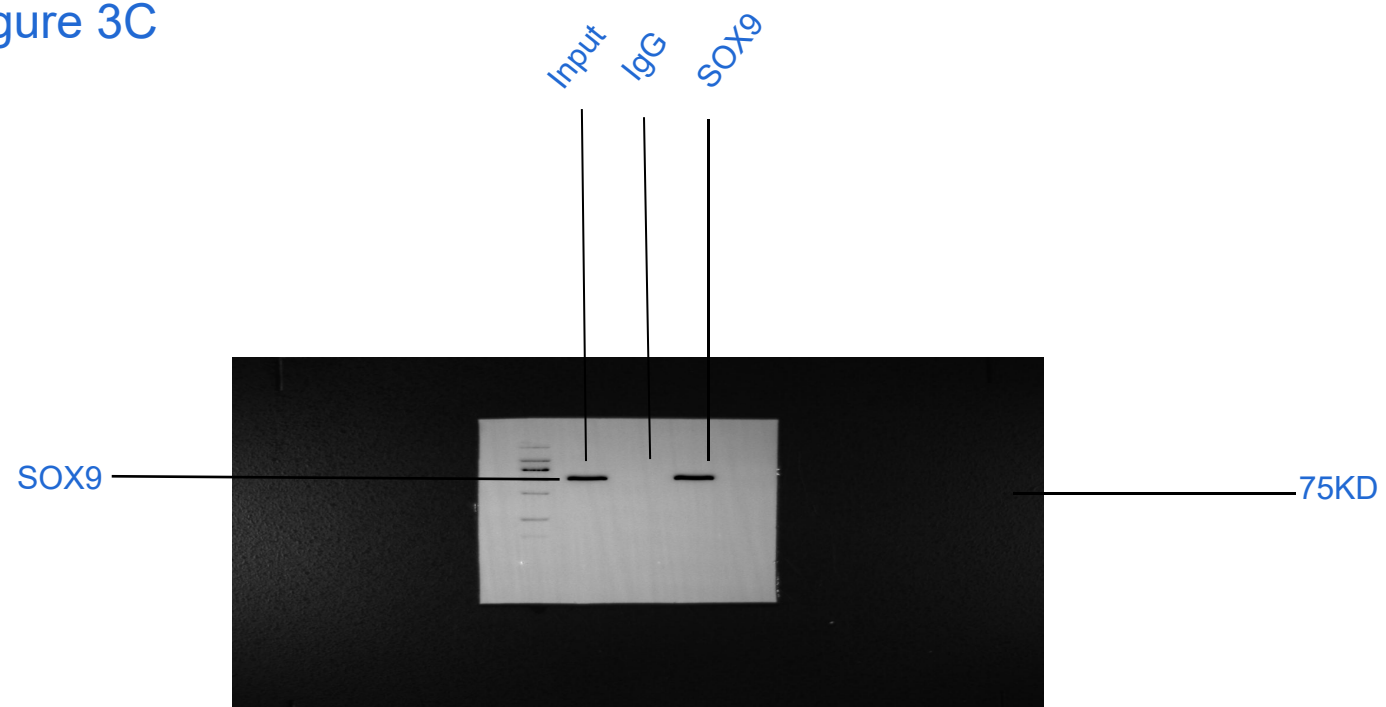

Figure 3F

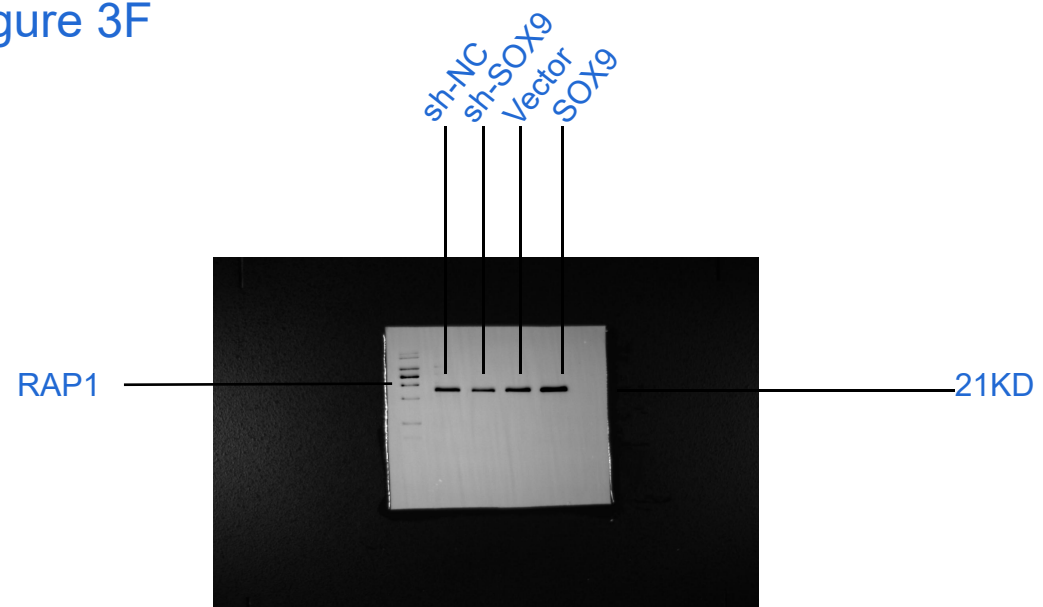

Figure 3F

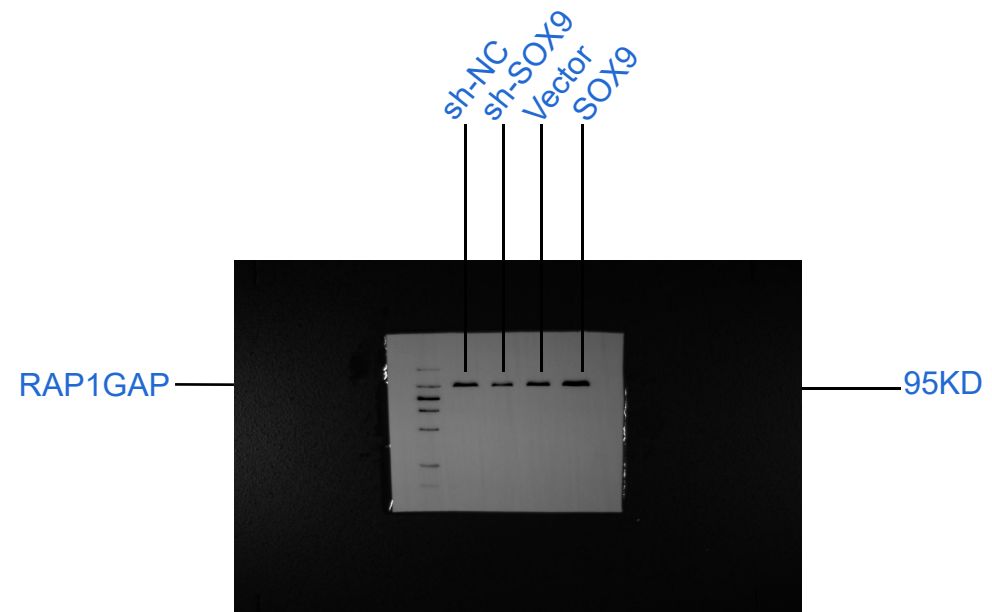

Figure 3F

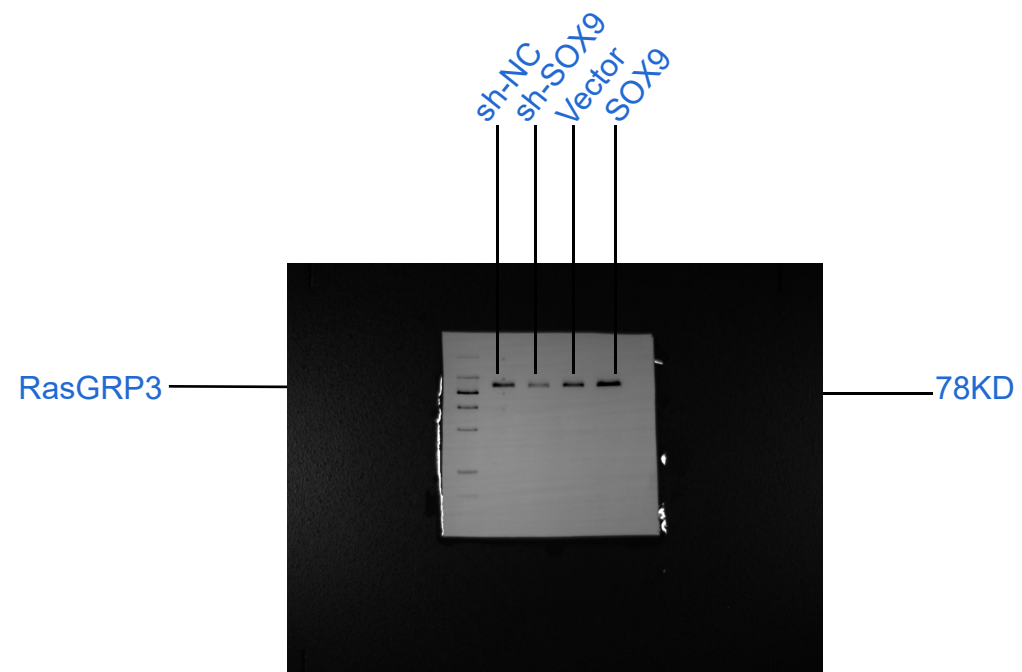

Figure 3F

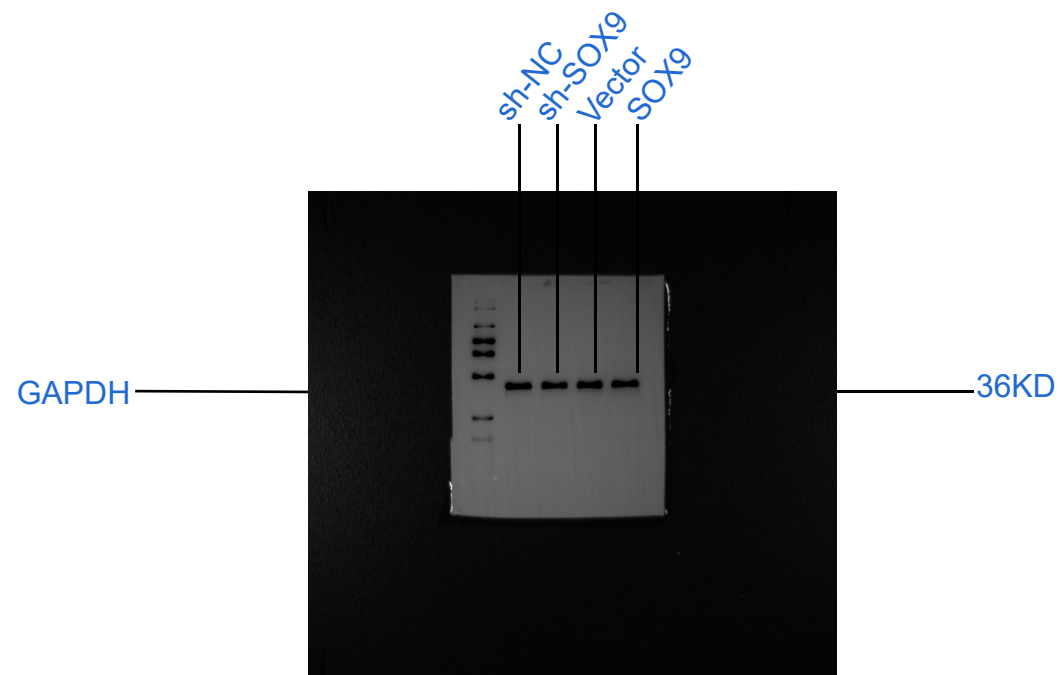

Figure 4C

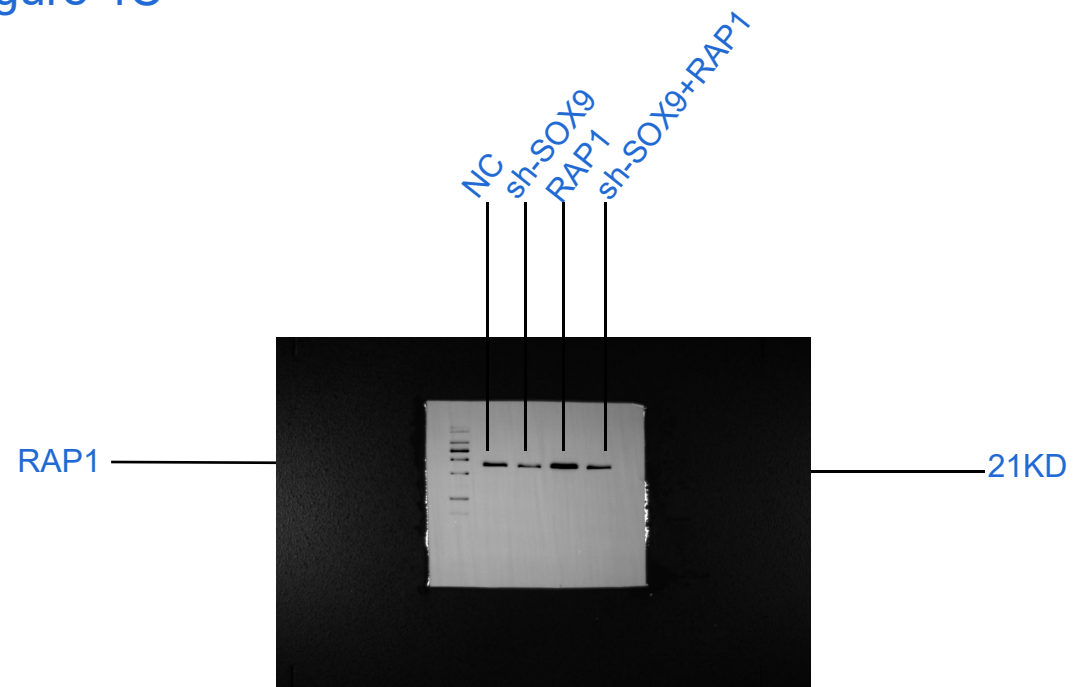

Figure 4C

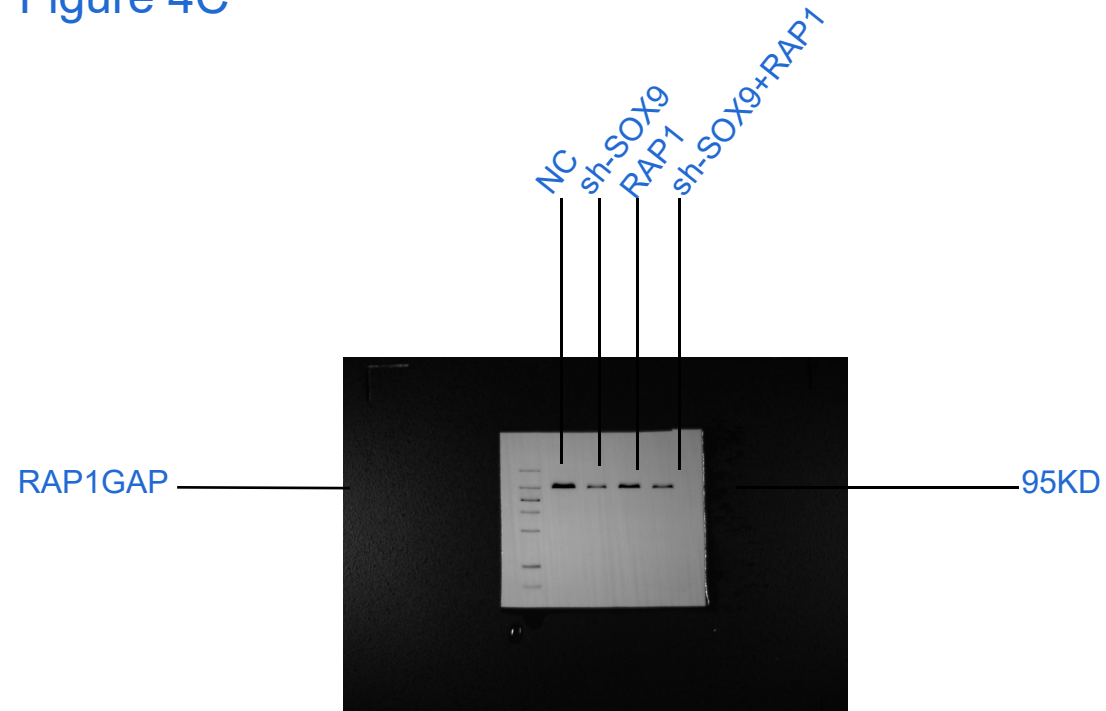

Figure 4C

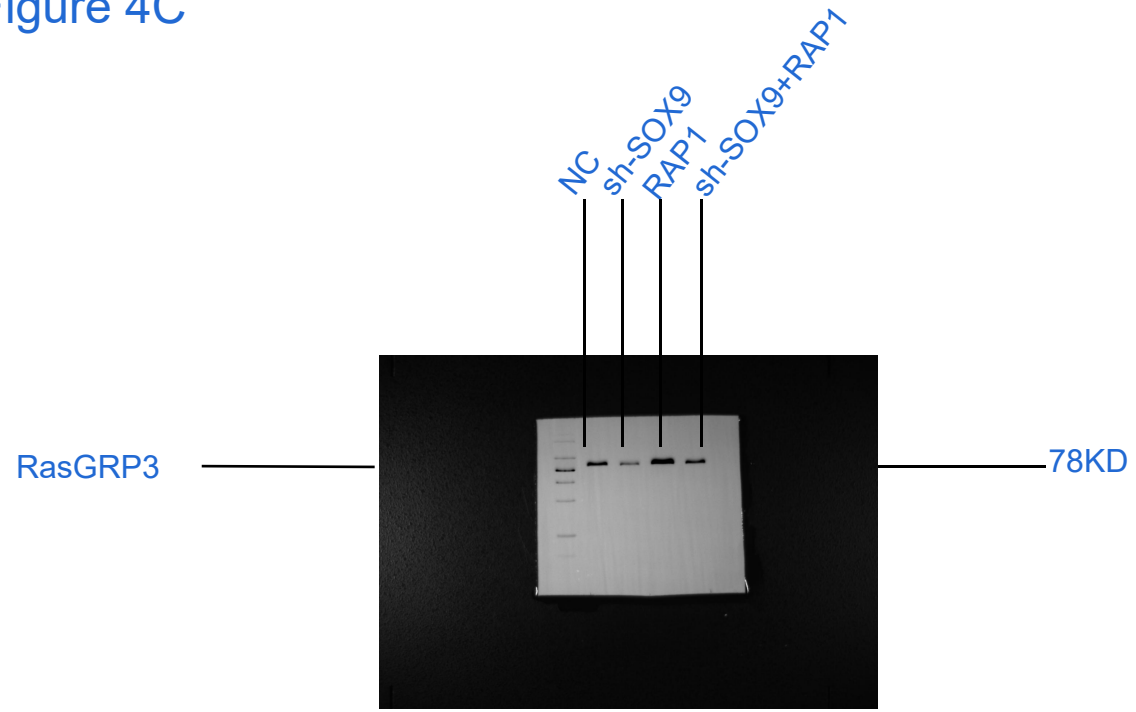

Figure 4C

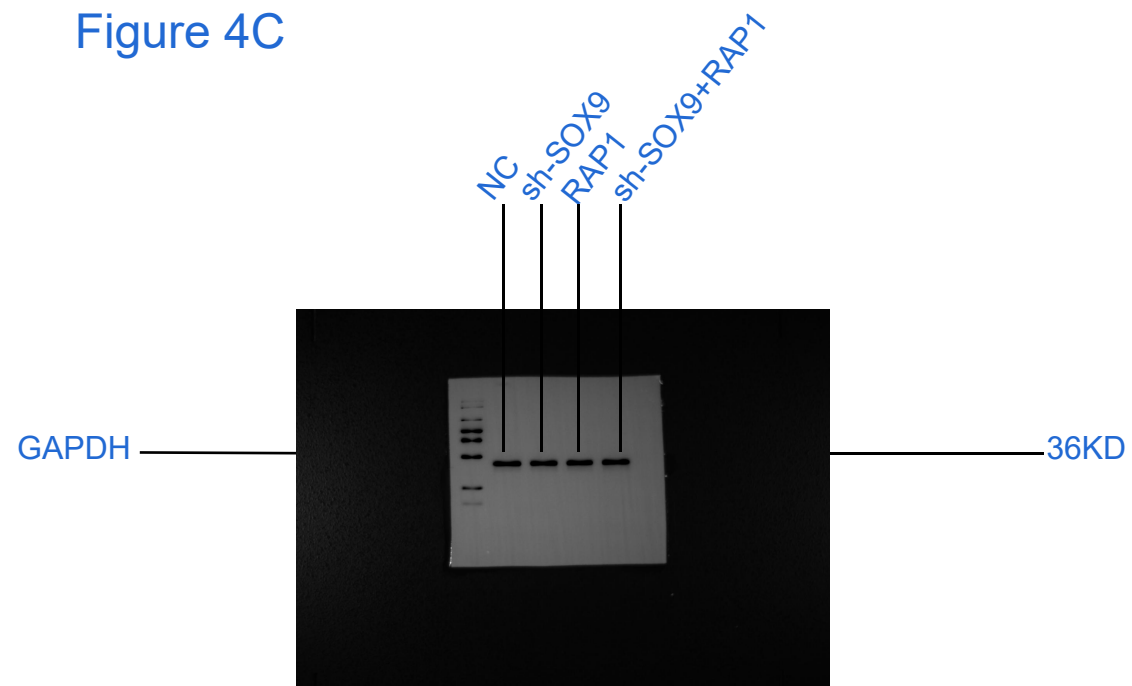

Supplement: Supplementary file 1 — Supplementary Material 1 [file 12890_2023_2740_MOESM1_ESM.pdf]
